# Neural correlates of distance-related functional reorganization in obsessive compulsive disorder

**DOI:** 10.1139/jpn-2026-0057

**Published:** 2026-08-26

**Authors:** Baohong Wen, Zihan Li, Keke Fang, Zijun Liu, Yufei Shen, Ya Tian, Jin Sun, Liang Liu, Xiaopan Zhang, Kangkang Xue, Ruiping Zheng, Huirong Guo, Yarui Wei, Yong Zhang, Shaoqiang Han

**Affiliations:** ^a^ Department of Magnetic Resonance Imaging, 191599 the First Affiliated Hospital of Zhengzhou University, China; ^b^ Engineering Technology Research Center for detection and application of brain function of Henan Province, China; ^c^ Engineering Research Center of medical imaging intelligent diagnosis and treatment of Henan Province, China; ^d^ Key Laboratory of magnetic resonance and brain function of Henan Province, China; ^e^ Key Laboratory of brain function and cognitive magnetic resonance imaging of Zhengzhou, China; ^f^ Key Laboratory of Imaging Intelligence Research medicine of Henan Province, China; ^g^ Henan Engineering Research Center of Brain Function Development and Application, China; ^h^ Department of Pharmacy, The Affiliated Cancer Hospital of Zhengzhou University & Henan Cancer Hospital, Zhengzhou 450008, China; ^i^ Department of Psychiatry, the First Affiliated Hospital of Zhengzhou University, China

**Keywords:** fMRI, functional connectivity strength, OCD, excitatory–inhibitory ratio, distance

## Abstract

**Background:**

While analyzing distance-dependent functional connectivity strength (FCS) yields valuable information about brain activity, whether the FCS alterations found in obsessive-compulsive disorder (OCD) are linked to inter-regional anatomical distance is still unknown. This study investigated distance-associated functional reorganization in OCD and its associations with clinical assessment data and neurotransmitter receptor/transporter densities.

**Methods:**

To quantify FCS across global, long-range, and short-range scales, we analyzed resting-state functional MRI data from 99 OCD patients and 104 matched healthy controls (HCs), followed by a post hoc exploratory seed-based functional connectivity (FC) analysis to further characterize the key brain regions that may contribute to the observed FCS abnormalities. Group differences and correlations with clinical measures (symptom severity, illness duration, and age of onset) and positron emission tomography-derived neurotransmitter receptor/transporter densities were examined.

**Results:**

Compared to HCs, OCD patients exhibited reduced long-range FCS in the bilateral fusiform and parahippocampal gyri, primarily driven by weakened connections with frontal, superior/middle temporal, precuneus, and cingulate regions. Short-range FCS was increased in the right dorsolateral prefrontal cortex but decreased in the precentral gyrus and middle cingulate cortex, mainly due to altered local connections involving the middle frontal gyrus, precentral gyrus, precuneus, and cerebellum. These alterations were robust in cross-validation. No significant correlations were found with clinical measures. However, both long- and short-range FCS changes correlated significantly with neurotransmitter receptors/transporter densities, particularly the excitatory–inhibitory ratio profile.

**Limitations:**

Neurotransmitter receptor/transporter maps were derived from publicly available datasets, which may not capture individual variation in receptor density.

**Conclusion:**

This study highlights distance-dependent functional connectivity reorganization in OCD and its neurochemical underpinnings, providing new insights into the disorder's pathophysiology.

## Introduction

Obsessive-compulsive disorder (OCD), defined by the presence of intrusive thoughts and/or repetitive compulsive behaviors, affects an estimated 1.5% to 2.7% of adults during their lifetime.^1^ Although relatively common, the precise neurobiological mechanisms underlying this condition are not yet fully elucidated. Contemporary neuroimaging research, particularly using resting-state functional magnetic resonance imaging (fMRI), has progressively associated the origins of psychiatric conditions like OCD with dysregulation in extensive, interacting neural networks.^2^^,^^3^

Individuals with OCD frequently demonstrate atypical functional connectivity (FC) patterns across various brain systems, indicative of impaired functional integration.^4^^,^^5^ Documented anomalies include heightened FC linking visual processing areas with frontostriatal circuits, amplified connectivity inside the bilateral default mode network, and excessive connectivity between the lateral parietal cortex and the precuneus. Such FC disturbances are considered possible indicators of psychopathology and may forecast responses to treatment.^6–8^ Conventional FC analyses, however, typically depend on pre-selected regions of interest, which can introduce bias and constrain whole-brain exploratory studies. To overcome this challenge, a data-driven technique termed functional connectivity strength (FCS) mapping has been introduced. This voxel-level approach calculates the cumulative connectivity of each voxel with all others, allowing for an assumption-free evaluation of the spatial distribution and functional significance of brain regions across the entire connectome.^9^^,^^10^ Consequently, FCS enables a comprehensive, hypothesis-neutral examination of whole-brain FC abnormalities.^11^ This method has proven effective in revealing functional reorganization profiles in several disorders, such as depression,^12^ Parkinson's disease,^11^ and OCD.^13^

Growing research indicates that the strength of FC is often constrained by the anatomical separation between brain regions.^10^^,^^14^ Accordingly, FCS can be categorized into short-range (sFCS) and long-range (lFCS) components based on distance thresholds, which are believed to respectively reflect local functional specialization and global network integration.^15^^,^^16^ This distance-specific analytic framework has uncovered FCS changes in conditions including schizophrenia,^17^ attention-deficit/hyperactivity disorder,^18^ and bipolar disorder.^19^ Nevertheless, existing FCS investigations in OCD have not systematically examined how anatomical distance might shape these functional alterations.^13^^,^^20^ Therefore, it remains to be determined whether FCS abnormalities in OCD patients follow a distance-dependent pattern.

Neurotransmitter systems, which critically regulate brain structure and function, are deeply implicated in the pathophysiology of psychiatric disorders like OCD.^21–24^ Neuroimaging evidence suggests that both structural and functional brain deviations correlate with regional distributions of neurotransmitter receptors.^25–30^ Importantly, the blood oxygenation level-dependent (BOLD) signal measured in fMRI is largely determined by the local equilibrium between excitatory and inhibitory synaptic activity.^31^ Excitatory glutamatergic transmission directly affects regional oxygen metabolism and BOLD contrast,^27^^,^^28^ whereas inhibitory GABAergic interneurons modulate the signal indirectly via feedback inhibition.^31^ Prior studies have connected FC irregularities to specific neurotransmitter pathways.^32^^,^^33^ For instance, disrupted FC in major depressive disorder has been linked to GABA and glutamate system dysfunction.^33^ In OCD, proton magnetic resonance spectroscopy studies propose that an excitatory–inhibitory (E/I) imbalance is a key pathophysiological factor.^34^^,^^35^ Clarifying the molecular correlates of functional brain abnormalities could thus deepen our comprehension of OCD and guide more precise treatment development.^36^

The current study sought to examine distance-dependent functional reorganization in OCD by applying the FCS method. Resting-state fMRI data were acquired from 99 OCD patients and 104 demographically matched healthy controls (HCs). We calculated and compared global, long-range, and short-range FCS maps for each group. Informed by prior reports of FC alterations in OCD,^13^^,^^20^ we hypothesized that patients would demonstrate significant distance-specific FCS abnormalities. Additionally, we performed a distance-dependent FC analysis seeded at the peaks of significant FCS differences to identify brain regions contributing to these alterations. Finally, we examined the associations between FCS abnormalities, neurotransmitter receptor/transporter densities, and clinical characteristics.

## Methods

### Participants

The protocol for this study was conducted in full compliance with the Declaration of Helsinki and received formal approval from the Research Ethics Committee of the First Affiliated Hospital of Zhengzhou University (Approval No. 2018-KY-88).

Prior to their enrollment, written informed consent was acquired from every participant. We enrolled 99 drug-naive patients experiencing their first episode of OCD from the psychiatric outpatient clinic of the same hospital. The diagnosis for each patient was independently verified by two senior psychiatrists according to Diagnostic and Statistical Manual of Mental Disorders, Fifth Edition (DSM-5) criteria. Symptom severity was quantified using the Yale-Brown Obsessive Compulsive Scale (Y-BOCS).^37^ Additionally, depressive and anxiety symptoms were assessed using the 24-item Hamilton Depression Rating Scale (HAMD)^38^ and the 14-item Depression Hamilton Anxiety Rating Scale (HAMA),^39^ respectively. Exclusion criteria for the patient group comprised: a history of neurological diseases or brain injury, comorbid psychiatric or psychotic disorders. 104 HCs of Han Chinese ethnicity and right-handed were recruited from the local community through advertisements. The HCs were interviewed using for DSM-5 Non-Patient version to exclude psychiatric disorders. None of the HCs had a history of serious medical or neuropsychiatric illness, nor a family history of major psychiatric or neurological disorders in their first-degree relatives. In addition, all participants met inclusion criteria to minimize potential confounding factors. These criteria included no use of analgesic or sedative medications within the past month, no history of substance abuse, brain tumors, head trauma, neurological surgery, or major systemic diseases, no MRI contraindications, and no detectable structural brain abnormalities on conventional MRI.

### Scan acquisition

Participants underwent resting-state fMRI scanning under standard instructions: lying supine with eyes closed, maintaining wakefulness, and refraining from directed mental activity. Over an 8 min session, 242 consecutive whole-brain functional images were captured using a gradient echo echo-planar imaging (GRE-EPI) sequence. The imaging protocol was defined by the following parameters: repetition time (TR) = 2000 ms, echo time (TE) = 30 ms, flip angle = 90°, field of view = 220 mm × 220 mm. The brain volume was sampled with 32 axial slices, yielding a voxel resolution of 3.4 × 3.4 × 4 mm^3^.

### Imaging data processing

Preprocessing of the functional imaging data was conducted within the SPM framework using the DPABI toolbox (http://rfmri.org/dpabi).^40^^,^^41^ The pipeline commenced with the removal of the initial 10 volumes to achieve signal stabilization. This was followed by slice timing correction and head motion realignment. Functional images were then normalized to the MNI EPI template and resampled to an isotropic 3 mm^3^ voxel size. Participants exhibiting head motion exceeding 3.0 mm in translation or 3° in rotation were to be excluded; however, no subject in this cohort met this criterion. Several procedures were implemented to mitigate and assess the potential effects of head motion on our results (see Discussion). Subsequent steps included spatial smoothing with a 6 × 6 × 6 mm^3^ full width at half maximum Gaussian kernel, detrending, and temporal band-pass filtering (0.01–0.1 Hz). To mitigate non-neuronal signals, a nuisance regression model was applied, incorporating the Friston 24-parameter model,^42^ along with signals from white matter and cerebrospinal fluid. Notably, global signal regression was omitted based on evidence that global signal fluctuations may hold neurobiological relevance in psychiatric conditions.^43–45^ Spike artifacts were corrected using the AFNI tool 3dDespike (http://afni.nimh.nih.gov/afni),^46^ which detects outliers via the median absolute deviation method and interpolates them using a third-order spline. Finally, mean frame-wise displacement (FD) was computed for each participant as a quantitative measure of head motion for subsequent group-level analysis.^47–50^

### Distance-dependent FC strength analysis

For each pair of voxels, FC was quantified by calculating Pearson's correlation between their time series. The FCS for a given voxel was then derived by summing its correlation coefficients with all other voxels, representing its total connectivity within the network. To minimize the influence of spurious or noise-driven correlations and given of the ambiguous biological interpretation of negative correlations in resting-state fMRI, only connections with correlation coefficients greater than *r* = 0.2 were retained for analysis. This threshold has been widely used in previous studies.^51^^–^^53^ We also computed the FCS values using *r* > 0.25 to ensure that our results were independent from the selection of thresholds.^54^ Voxel-wise connections were categorized as sFCS or lFCS based on a 75 mm distance criterion, with the anatomical distance between 2 voxels defined as Euclidean distance (in mm) between the stereotaxic coordinates.^55^ In addition to sFCS and lFCS, the global FCS (gFCS) was also calculated. All FCS maps were subsequently transformed into *Z*-scores to enable group-level statistical comparisons.

Group differences in gFCS, lFCS, and sFCS between OCD patients and HCs were examined in SPM12 (http://www.fil.ion.ucl.ac.uk/spm) using two-sample *t*-tests, with age, sex, education, and mean FD included as nuisance covariates. Statistical significance was assessed using Gaussian Random Field (GRF) correction for multiple comparison correction (voxel *p* < 0.005, cluster *p* < 0.05).

To assess the robustness of group differences and ensure that FCS abnormalities were not driven by a subset of patients, an internal cross-validation procedure was conducted. In each iteration, 90% of OCD patients were randomly selected, and FCS differences with the HC group were recomputed via two-sample *t*-tests. Spatial correlations were calculated between the unthresholded *t*-maps derived from the 90% subsample and those based on the full OCD group. Repeating (100 times) this process allowed us to test the stability of the FCS abnormalities across different patient subsets.

### Voxel-wise distance-dependent FC analysis

Although we have identified FCS abnormalities in MDD, we have no idea brain regions contributing to the observed abnormalities. Thus, we performed a post hoc exploratory seed-based FC analyses to further characterize the key brain may contribute to the observed FCS abnormalities. We identified peak coordinates of significant FCS (lFCS/sFCS) group differences. Second, a spherical region (6 mm radius) was defined around each peak voxel to serve as a seed. For each seed, Pearson's correlation was calculated between the average time series of the seed and all other voxels. The resultant correlation maps underwent Fisher's *r*-to-*z* transformation to improve normality, yielding *Z*-score maps. To maintain consistency with the FCS classification, FC maps from sFCS peaks were restricted to short-range connections, and those from lFCS peaks were restricted to long-range connections. Group-level comparisons were then performed between OCD and HC groups for each seed using two-sample *t*-tests, with statistical significance determined by GRF correction (voxel-level *p* < 0.005, cluster-level *p* < 0.05).

### Associations between FCS abnormalities and clinical features

To explore relationships between altered FCS and clinical characteristics, we extracted the mean FCS values (lFCS/sFCS) from a 6 mm spherical ROI centered at each peak coordinate showing significant group differences. Pearson's correlations analyses were conducted between these FCS values and the total score of Y-BOCS, its subscale scores (obsession and compulsion), HAMD and HAMA scores, age of onset, and illness duration. Statistical significance was set at *p* < 0.05 with false-discovery-rate correction (Benjamini–Hochberg procedure).

Additionally, we calculated Pearson's correlations between these FCS values and mean FD to further assess the effects of head motion.

### Neurotransmitter receptor/transporter distributions in relation to altered FCS patterns

To investigate the molecular underpinnings of FCS alterations in OCD, we examined the relationship between regional densities of neurotransmitter receptor/transporter and group differences in lFCS and sFCS. Neurotransmitter receptor/transporter densities were derived from a positron emission tomography (PET)-based atlas.^56^ This atlas provides normalized density maps for a comprehensive set of neurotransmitter systems, including serotonin (5HT_1A_,^57^ 5HT_1B_,^57–63^ 5HT_2A_,^64^ 5HT_4_,^65^ 5HT_6_,^66^^,^^67^ 5HTT^65^), norepinephrine (α_4_β_2_,^63^^,^^68^ M_1_,^69^ VAChT,^70^^,^^71^), cannabinoid (CB_1_^72–75^), dopamine (D_1_,^76^ D_2_,^77–80^ DAT^81^), GABA (GABA_a_^82^), histamine (H_3_^83^), glutamate (mGluR_5_,^84^^,^^85^ NMDA^86^^,^^87^), opioid (MOR^88^), and norepinephrine (NET^89–92^). All PET images were averaged across participants, spatially normalized to the MNI-ICBM 152 nonlinear 2009 template, and parcellated into 268 brain regions. Regional receptor/transporter densities were *Z*-scored prior to analysis.^23^^,^^56^

For each region, we obtained the mean unthresholded *t*-statistic values from the comparison of OCD patients and HCs (from the voxel-wise FCS analysis). A multilinear regression model was then established to assess the association between the receptor/transporter profiles (predictors) and the regional *t*-statistics (outcome). To determine statistical significance, we generated 1000 surrogate brain maps preserving spatial autocorrelations and recalculated the regression each time to generate a null distribution for significance testing (*p*_permutation_) using the BrainSMASH.^93^ To quantify the proportionate contribution of each neurotransmitter system to the model, we calculated the normalized regression coefficients for each receptor/transporter. Predictors with higher absolute coefficient values were interpreted as having a greater influence on explaining the FCS abnormalities.

Additionally, we computed an indirect PET receptor–density-based proxy for the excitation/inhibition balance (E/I ratio) for each brain region that defined as the ratio between the average density of excitatory versus inhibitory receptors.^29^^,^^30^ Excitatory receptors include 5HT_2A_, 5HT_4_, 5HT_6_, D_1_, α_4_β_2_, M_1_, and NMDA. Inhibitory receptors include 5HT_1A_, 5HT_1B_, CB_1_, D_2_, H_3_, MOR, and GABA_a_. Next, we computed spatial correlations between the regional E/I ratio distribution and the lFCS/sFCS abnormality maps (derived from the unthresholded *t*-statistics of the OCD vs. HC comparison). The relevance of these observed associations was assessed via using the BrainSMASH (1000 iterations).

## Results

### Demographic characteristics

No significant differences were observed in age or sex distribution between the OCD group and HCs. However, the OCD group had significantly fewer years of education than HCs (*p* < 0.001). Mean FD also did not differ significantly between groups (*p* = 0.392), indicating comparable head motion during scanning. Demographic and clinical characteristics are summarized in Table 1.

**Table 1.** Demographic and clinical characteristics.

	OCD (*N* = 99)	HCs (*N* = 104)	*p*
Age (years), mean (SD)	23.16 (9.34)	23.14 (5.64)	0.987^a^
Male, No. (%)	52 (53.06%)	51 (49.04%)	0.619^b^
Educational level (years), mean (SD)	11.95 (3.04)	15.21 (3.17)	<0.001^a^
Illness duration (months), mean (SD)	41.93 (10.53)	–	–
Age of onset (years)	16.50 (10.18)	–	–
Y-BOCS, mean (SD)	21.73 (6.91)	–	–
Obsession, mean (SD)	11.64 (3.54)	–	–
Compulsion, mean (SD)	10.28 (4.31)	–	–
HAMD	34.80 (15.20)	–	–
HAMA	23.55 (13.49)	–	–
Mean FD (mm), mean (SD)	0.11 (0.05)	0.13 (0.14)	0.392^a^

**Note:** OCD, obsessive compulsive disorder; HC, healthy control; Y-BOCS, Yale–Brown obsessive compulsive scale; HAMD, Hamilton Depression Rating Scale; HAMA; Hamilton Anxiety Rating Scale; mean FD, mean frame-wise displacement.

^a^Two-sample *t* test.

^b^χ^2^ test.

### Altered FCS in OCD

**Fig. 1.**
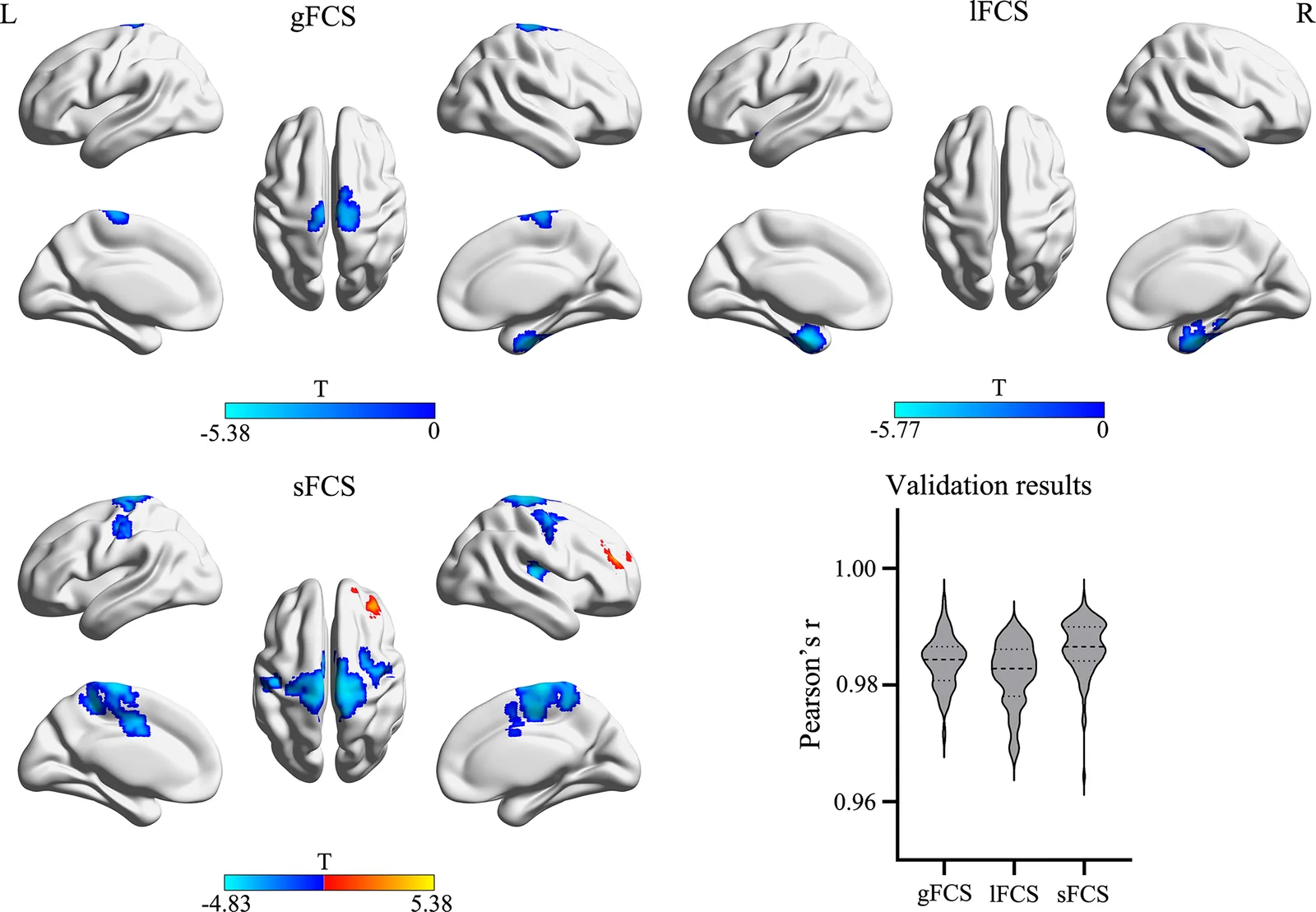
Distance-dependent FCS abnormalities in OCD. Brain maps showing regions with significantly altered global (gFCS), long-range (lFCS), and short-range (sFCS) functional connectivity strength in OCD compared to HCs. Violin plots showing distributions of spatial correlations (Pearson's *r*) from internal cross-validation, indicating stability of FCS alterations across subsamples.

Compared to HCs, OCD patients exhibited significant alterations in gFCS, lFCS, and sFCS. Decreased gFCS was found in the right fusiform and parahippocampa gyrus, and precentral gyrus (Fig. 1; Table S1). Decreased lFCS was found in the bilateral fusiform and parahippocampa gyrus (Fig. 1; Table S2). sFCS alterations included increased sFCS in the right dorsolateral prefrontal cortex (DLPFC), and decreased sFCS in the precentral gyrus extending to medial frontal gyrus and middle cingulate cortex (Fig. 1; Table S3). All findings survived GRF correction for multiple comparisons with thresholds set at *p* < 0.005 for voxel-level and *p* < 0.05 for cluster-level. Even using another threshold in the calculation of FCS values (*r* > 0.25), these differential patterns of FCS remained largely unchanged (Fig. S1).

To assess the robustness of these results, internal cross-validation analysis was conducted. Across 100 iterations using 90% of OCD patients, high spatial correlations (mean ± SD) were observed between the subsampled and full-group *t*-maps for gFCS (0.984 ± 0.004), lFCS (0.982 ± 0.006), and sFCS (0.987 ± 0.005), demonstrating strong reproducibility (Fig. 1).

### Voxel-wise and distance-dependent FC analysis

Subsequently, post hoc exploratory seed-based FC analyses were performed to identify the specific brain regions whose altered connectivity patterns underlay the observed FCS abnormalities. Two clusters identified in the gFCS analysis were largely encompassed by the lFCS and sFCS clusters, respectively (see Fig. 1). Quantitative spatial overlap analysis showed that 99.55% (219/220 voxels) of Cluster 1 overlapped with the lFCS cluster, and 98.21% (220/224 voxels) of Cluster 2 overlapped with the sFCS cluster, indicating a high degree of spatial correspondence between these regions. This spatial overlap indicates that the gFCS abnormalities were primarily driven by the corresponding lFCS and sFCS alterations. Thus, subsequent analyses focused on these two distance-dependent FCS types. Specifically, seeds were located at [30, –12, –33] and [–27, –3, –33] for lFCS abnormalities, and at [39, 48, 27] and [–15, –36, 54] for sFCS abnormalities.

**Fig. 2.**
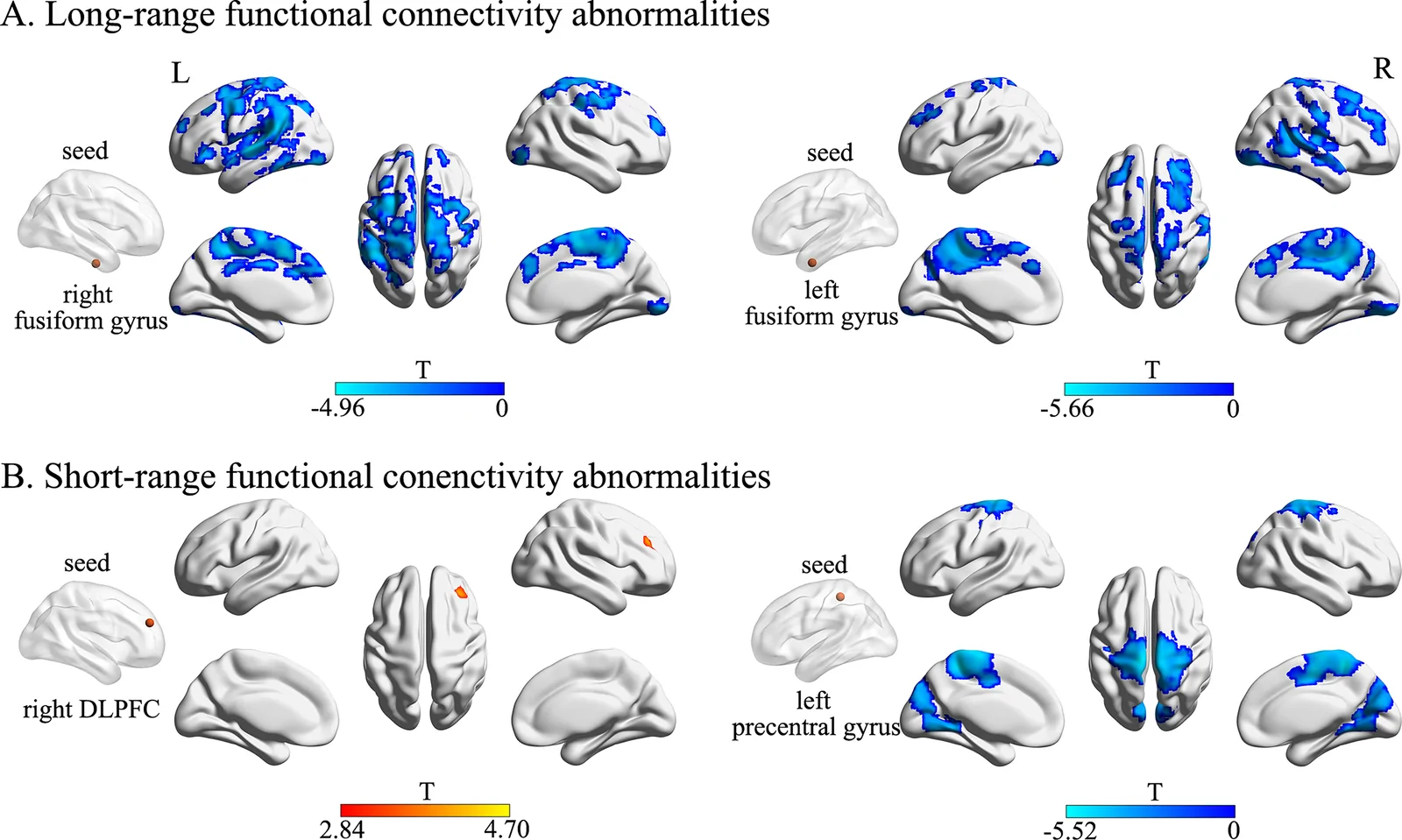
Distance-dependent functional connectivity abnormalities in OCD. Distance-dependent functional connectivity abnormalities seeded at peaks of clusters showing long- or short-range FCS abnormalities in OCD compared with HCs. Abbreviation: DLPFC, dorsolateral prefrontal cortex.

Patients with OCD had significantly reduced long-range FC between the right fusiform gyrus ([30, –12, –33]) and widespread frontal regions, including the medial, middle, and superior frontal gyri, along with the precentral and postcentral gyri and precuneus (Fig. 2; Table S4). Similarly, decreased long-range FC was observed between the left fusiform gyrus ([–27, –3, –33]) and the superior and middle temporal gyri, precuneus, middle frontal gyrus, and cingulate gyrus (Fig. 2; Table S4).

Regarding the sFCS abnormalities, patients with OCD exhibited increased short-range FC between the right middle frontal gyrus ([39, 48, 27]) and the medial frontal gyrus (Fig. 2; Table S5). In contrast, decreased short-range FC was observed between the left precentral gyrus ([–15, –36, 54]) and the precuneus, middle cingulate gyrus, and anterior lobe of the cerebellum (Fig. 2; Table S5).

All results remained significant after GRF correction for multiple comparisons with thresholds set at *p* < 0.005 for voxel-level and *p* < 0.05 for cluster-level.

### Associations between altered FCS and clinical features

We next investigated the associations between altered FCS (lFCS/sFCS) and clinical features. No significant correlations were found between alterations in FCS and clinical measures, including the total score of Y-BOCS, its subscale scores (obsession and compulsion), HAMD and HAMA scores, age of onset, and illness duration (all uncorrected *p* values > 0.05). The correlation coefficients and the corresponding *p* are available in the Table S6.

Additionally, we also did not find any significant correlations between alterations in FCS and mean FD, further excluding the effects of head motion.

### Neurotransmitter receptor/transporter distributions in relation to altered FCS patterns

To investigate the relationship between these FCS alterations and neurotransmitter receptor/transporter distributions, we assessed the spatial correspondence between the observed differences in lFCS and sFCS in OCD and publicly available PET maps of neurotransmitter receptors and transporters. A multilinear regression model was developed using the spatial patterns of neurotransmitter receptor/transporter as predictors, with regional FCS differences serving as the dependent variable. The model demonstrated significant explanatory power for both lFCS and sFCS abnormalities, with adjusted *R*^2^ values of 0.49 (*F*-statistic (268, 248) = 14.30) and 0.43 (*F*-statistic (268, 248) = 11.70) for lFCS and sFCS abnormalities, respectively (*P*_permutation _< 0.001 for both).

**Fig. 3.**
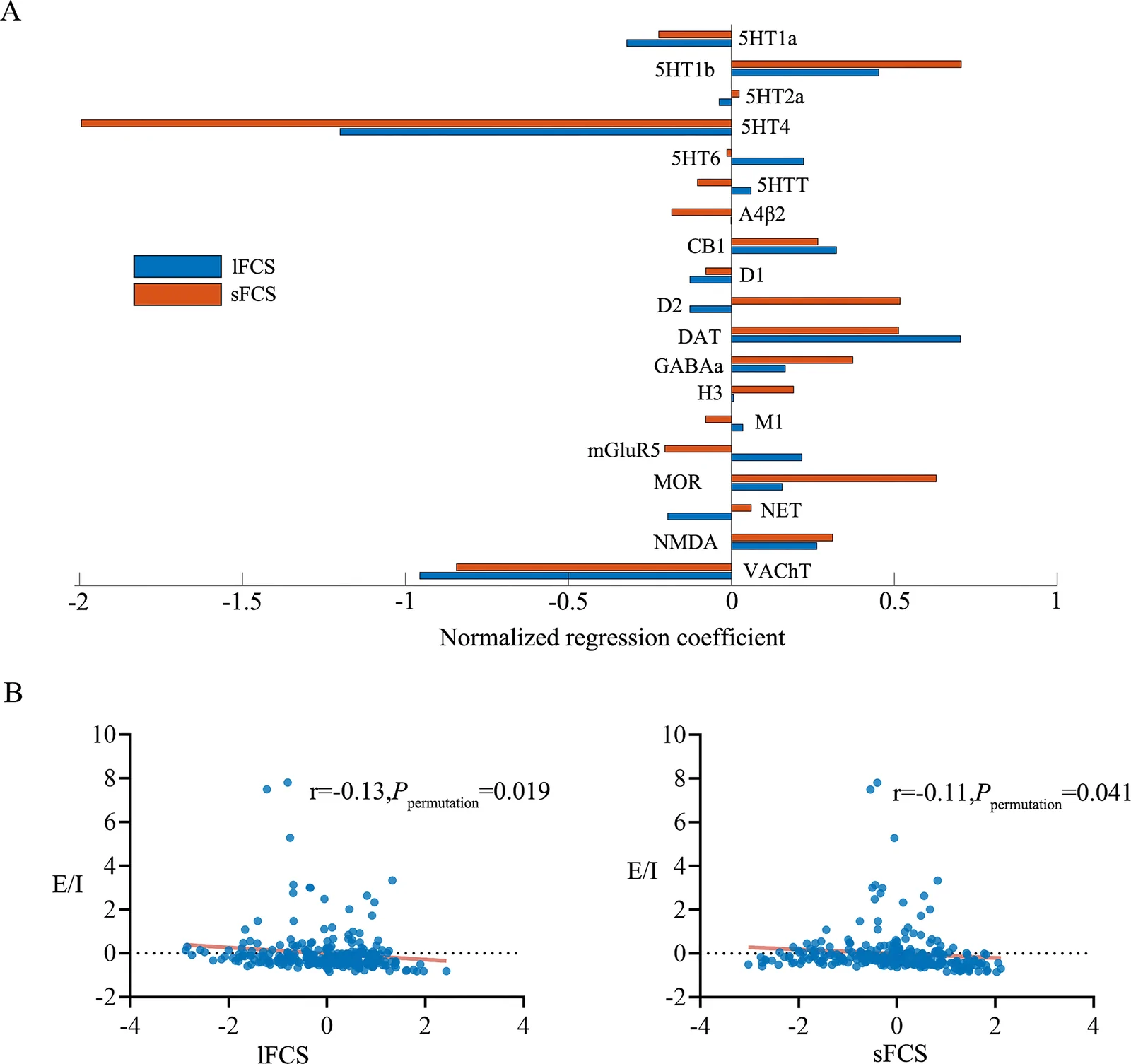
Correlations between FCS abnormalities and neurotransmitter receptor/transporter profiles. (A) Normalized regression coefficients from the multilinear regression model depicting the relative contribution of each receptor/transporter to lFCS and sFCS abnormalities. (B) Spatial correlations between regional lFCS/sFCS abnormalities and the excitatory–inhibitory (E/I) ratio.

Among all receptors, 5HT_4_ exhibited the largest absolute coefficient, followed by VAChT, 5HT_1b_, and DAT, suggesting their key contributions to both lFCS and sFCS alterations in OCD. Additionally, 5HT_6_ and H_3_ were identified as particularly important for lFCS and sFCS abnormalities, respectively (Fig. 3A).

We also assessed the relationship between an indirect PET receptor–density-based proxy for the excitation/inhibition balance (E/I ratio) and FCS abnormalities. The distribution pattern of FCS differences showed a negative correlation with the regional E/I ratio (lFCS: *r* = −0.13, *P*_permutation_ = 0.019; sFCS: *r* = −0.11, *P*_permutation_ = 0.041, Fig. 3B). To confirm that these results were not driven by extreme values, we repeated the analysis after excluding outlier regions—defined as regions with FCS or E/I ratio exceeding ±3 standard deviations from the mean (*n* = 6). The correlations remained significant after exclusion (lFCS: *r* = −0.13, *P*_permutation_ = 0.022; sFCS: *r* = −0.16, *P*_permutation_ = 0.011), confirming the robustness of these associations.

## Discussion

In the present study, we applied a distance-dependent FCS method was applied to thoroughly assess intrinsic functional changes in OCD. Relative to HCs, OCD patients exhibited significant distance-dependent FCS abnormalities, characterized by decreased lFCS in the bilateral fusiform and parahippocampal gyri, increased sFCS in the right DLPFC, and decreased sFCS in the precentral gyrus and middle cingulate cortex. Further distance-dependent FC analyses revealed that reduced lFCS was primarily driven by decreased long-range connectivity between the bilateral fusiform gyrus and widespread regions, including the middle frontal gyrus, precentral gyrus, precuneus, and temporal gyrus. Similarly, abnormal sFCS patterns in OCD were attributed to local FC changes involving the middle frontal gyrus, precentral gyrus, and precuneus. Moreover, both lFCS and sFCS abnormalities correlated significantly with the distributions of neurotransmitter receptor/transporter, especially the E/I ratio, which points to a molecular foundation for these functional alterations.

Consistent with prior neuroimaging findings, OCD patients in this study showed distributed FCS abnormalities. Previous studies have reported inconsistent FCS alterations in OCD, which could be attributed to the limited sample sizes and the insufficient of separation between short- and long-range connectivity. For example, Bu et al. reported no significant global FCS differences between patients with OCD who were drug-naive and HCs,^52^ whereas a different study observed altered sFCS in the precentral/postcentral gyrus and increased lFCS in the thalamus, inferior parietal lobule, and cerebellum, though using relatively lenient thresholds.^94^ Our study, utilizing the most extensive OCD cohort thus far and rigorous control of potential confounders (e.g., medication, comorbidities), identified robust FCS abnormalities that survived strict correction and cross-validation analysis, supporting their reproducibility. When decomposing global FCS into distance-dependent components, more specific alterations emerged. Patients with OCD exhibited decreased lFCS in the bilateral fusiform and parahippocampal gyri, and elevated sFCS in the right DLPFC, as well as lower sFCS in sensorimotor and cingulate regions. These results suggest spatially distinct disruptions in local and distributed network integration in OCD. Although we adopted a relatively lenient head-motion exclusion threshold to maximize the sample size, we conducted several complementary analyses to minimize and evaluate the potential impact of head motion on our results. First, despiking was applied to reduce the influence of motion-related outliers by replacing them with estimates derived from a third-order spline fit. Second, group comparisons showed no significant difference in mean FD between patients with OCD and healthy controls. This strategy is commonly used to mitigate the effects of head motion.^95^ Third, head motion was included as a nuisance covariate in the group-level analyses. Fourth, we further examined the relationship between FCS alterations and mean FD and found no significant correlations. Taken together, these results suggest that head motion is unlikely to have substantially influenced the observed findings. Additionally, these FCS abnormalities showed no significant correlations with symptom severity, illness duration, or age at onset, suggesting that they may represent trait-like markers of the disorder. Several factors likely contribute to this negative finding. First, FCS may primarily reflect stable, trait-like alterations in intrinsic brain network organization that persist beyond acute symptom fluctuations. Second, current clinical scales may lack the sensitivity to capture subtle neurobiological variability in connectivity, particularly in non-obsessional domains or during remission. Third, the observed FCS changes could represent downstream consequences of OCD-related neural vulnerability rather than direct correlates of current symptom intensity. Future longitudinal studies with larger samples and multimodal imaging are warranted to clarify the temporal dynamics and clinical relevance of these network alterations.

The fusiform gyrus exhibited reduced long-range connectivity with multiple cortical regions. This region participates in multiple functions, including visual organization, memory, and the processing of social information,^96–100^ and has been shown to exhibit structural and functional abnormalities in OCD.^96^^,^^101–105^ The observed reductions in fusiform-based long-range connectivity may underlie visual and perceptual processing deficits commonly reported in OCD.^106^ Similarly, the precentral and postcentral gyri, key regions for sensorimotor integration,^107^ showed decreased sFCS, suggesting reduced efficiency of sensorimotor information transmission in OCD.^108^^,^^109^ Previous findings indicate that lFCS in this region may serve as a neurobiological biomarker, capable of distinguishing OCD patients from HCs with high accuracy.^94^ Aligning with these findings, the post-hoc seed-based FC analysis results revealed that reduced lFCS in the fusiform gyrus were predominantly contributed by its long-range FC with distributed brain regions, including the frontal gyrus, precuneus, pre- and post-central gyrus, highlighting the region's role in large-scale functional abnormalities in OCD.^103^ The parahippocampa gyrus also exhibited decreased lFCS in patients with OCD. As a key hub of default-mode network, the parahippocampa gyrus involved in multiple cognitive processes, including visuospatial and episodic memory.^110^ Previous studies have reported structural and functional abnormalities in this region in OCD, which may be related to damage in reward and punishment processing in patients with OCD.^111^ These regional abnormalities are further supported by findings of decreased regional homogeneity in the parahippocampal gyrus in patients with OCD compared with healthy controls.^112^ In addition, the decreased long-range FCS may be associated with reduced average controllability and increased modal controllability in this region.^113^ In our prior study using the same cohort, we examined dynamic transitions between brain states using network control theory^113^ and observed decreased average controllability and increased modal controllability in the parahippocampal gyrus, which may underlie compulsive behaviors.^113^ In the current work, we extend these findings by quantifying distance-dependent lFCS and short-range FCS across the entire connectome, thereby providing an independent, whole-brain characterization of network architecture that complements the node-level controllability metrics. This approach not only confirms the parahippocampal gyrus as a key region with deficits in long-range connectivity and modal controllability, but also highlights the broader network-level implications for compulsive behaviors in OCD. By comparison, elevated sFCS in the right DLPFC, a critical node in the dorsal cognitive cortico–striato–thalamo–cortical circuit, may reflect inefficient local network organization related to response inhibition and cognitive control deficits in OCD.^114–116^ Although altered functional connections of this region have been widely reported, our results suggested that it predominantly exhibited short-range connectivity in patients with OCD.^117^

To explore potential molecular associations of FCS alterations, we examined spatial correlations between group-level FCS abnormality maps and normative neurotransmitter receptor/transporter maps derived from public PET datasets. After controlling for spatial autocorrelation and correcting for multiple comparisons, FCS abnormalities showed the strongest spatial associations with serotonergic receptor maps, particularly via 5-HT_4_, 5-HT_1_B, and 5-HT_6_ receptors, which are which is critically involved in both OCD pathophysiology and treatment response.^118^^,^^119^ In addition, FCS alterations were spatially correlated with an indirect PET receptor-density-based E/I proxy. This finding aligns with neurobiological models positing that OCD arises from hyperactivation within orbitofronto–striatal circuits, driven by disruptions in the equilibrium between direct/excitatory and indirect/inhibitory pathways.^120^ However, these findings should be interpreted cautiously, as they do not reflect individual-level neurotransmitter measurements in our participants and therefore cannot be taken as evidence of true receptor abnormalities or E/I imbalance in OCD. Rather, they suggest that the spatial distribution of FCS abnormalities may overlap with normative molecular architectures implicated in serotonergic signaling and excitation–inhibition-related receptor profiles. Thus, these results provide exploratory and indirect evidence for possible molecular associations with large-scale functional network alterations in OCD.

In summary, this study revealed distance-dependent FC alterations in OCD, characterized by reduced long-range and altered short-range integration across key cortical regions. These alterations were significantly correlated with neurotransmitter receptor profiles and E/I balance, yielding novel insight into the molecular basis of OCD-related large-scale network dysfunction. Our findings highlight the value of the distance-dependent FCS framework for bridging molecular, functional, and clinical dimensions of psychiatric disorders.

## Limitations

Several limitations should be acknowledged. First, although cross-validation verified the robustness of our findings, replication in independent datasets is warranted. A second limitation stems from the paucity of clinical information, which hindered detailed exploration of symptom–function relationships. Future studies should incorporate comprehensive clinical and cognitive assessments as well as information of environment exposures.^121^ Third, the seeds used in the seed-based FC analysis were selected from significant FCS group-difference peaks identified within the same dataset, which may introduce a degree of circularity. Moreover, causality cannot be established, and the limited availability of clinical information restricts interpretability. Therefore, these results should be interpreted as post hoc exploratory findings rather than independent validation or causal evidence. Nevertheless, the seed-based FC analysis was conducted to further characterize the connectivity patterns that may contribute to the observed FCS abnormalities. Fourth, neurotransmitter receptor/transporter maps were derived from publicly available datasets, which may not capture individual variation in receptor density. Fifth, only patients without other mental disorders comorbidities are recruited in this study; future studies can investigate whether our conclusion hold true in more heterogeneous samples. Sixth, the current study overlooked the temporal dynamics of the intrinsic brain activity; future studies can investigate the dynamic variability of distance-dependent FCS using methods such as sliding window approach.^95^ Finally, OCD patients in this study had fewer years of education than HCs; potential effects of educational background warrant further investigation.

## Data Availability

The data used in this study are under active use by the reporting laboratory and are available from the corresponding author upon request.

## Supplementary material

Supplementary Material 1 (DOCX/ 416 KB)
