# Supplementary material for: Neural correlates of distance-related functional reorganization in obsessive compulsive disorder
Source: J Psychiatry Neurosci. 2026 Aug 26;51:1–11. doi: 10.1139/jpn-2026-0057 (PMC13504623; doi:10.1139/jpn-2026-0057)
Supplement: Supplementary file 1 [file jpn-2026-0057suppla.docx]

**Figure S1**. Validation of distance-dependent FCS abnormalities in OCD (threshold r > 0.25). Brain maps showing regions with significantly altered global (gFCS), long-range (lFCS), and short-range (sFCS) functional connectivity strength in OCD compared to HCs.

**Table S1**. Altered gFCS in OCD

| Cluster | Size | Peak MNI | T | Including regions |
| --- | --- | --- | --- | --- |
| 1 | 224 | 30 -12 -33 | -5.38 | Right fusiform gyrus |
|  |  |  |  | Right Parahippocampa gyrus |
| 2 | 220 | 15 -24 75 | -3.92 | Bilateral precentral gyrus |

**Table S2**. Altered lFCS in OCD

| Cluster | Size | Peak MNI | T | Including regions |
| --- | --- | --- | --- | --- |
| 1 | 396 | 30 -12 -33 | -5.77 | Right Fusiform gyrus |
|  |  |  |  | Right Parahippocampa gyrus |
| 2 | 284 | -27 -3 -33 | -5.49 | Left Fusiform gyrus |
|  |  |  |  | Left Parahippocampa gyrus |

**Table S3**. Altered sFCS in OCD

| Cluster | Size | Peak MNI | T | Including regions |
| --- | --- | --- | --- | --- |
| 1 | 279 | 39 48 27 | 5.38 | Right dorsolateral prefrontal cortex |
| 2 | 1320 | -15 -36 54 | -4.83 | Bilateral precentral gyrus |
|  |  |  |  | Bilateral middle cingulate gyrus |

**Table S4**. Altered long-range functional connectivity in OCD

| Seed | Cluster | Size | Peak MNI | T | Including regions |
| --- | --- | --- | --- | --- | --- |
| 30, -12, -33 | 1 | 7890 | -51 -45 39 | -4.96 | Bilateral medial frontal gyrus |
|  |  |  |  |  | Bilateral superior frontal gyrus |
|  |  |  |  |  | Bilateral middle frontal gyrus |
|  |  |  |  |  | Bilateral precuneus |
|  |  |  |  |  | Bilateral precentral gyrus |
|  |  |  |  |  | Bilateral postcentral gyrus |
| -27 -3 -33 | 1 | 2914 | 45 -87 -15 | -4.84 | Bilateral superior temporal gyrus |
|  |  |  |  |  | Right middle temporal gyrus |
|  |  |  |  |  | Right inferior occipital gyrus |
|  | 2 | 3927 | 0 -39 51 | -5.67 | Bilateral precuneus |
|  |  |  |  |  | Bilateral middle frontal gyrus |
|  |  |  |  |  | Bilateral cingulate gyrus |

**Table S5**. Altered short-range functional connectivity in OCD

| Seed | Cluster | Size | Peak MNI | T | Including regions |
| --- | --- | --- | --- | --- | --- |
| 39 48 27 | 1 | 161 | 39 42 39 | 4.70 | Right middle frontal gyrus |
| -15 -36 54 | 1 | 1437 | -12 -48 -9 | -4.94 | Bilateral precuneus |
|  |  |  |  |  | Bilateral cuneus |
|  | 2 | 2718 | -6 -45 57 | -5.52 | Precuneus |
|  |  |  |  |  | Postcentral gyrus |
|  |  |  |  |  | Bilateral precentral gyrus |
|  |  |  |  |  | Bilateral medial frontal gyrus |
|  |  |  |  |  | Bilateral cingulate gyrus |

**Table S6**. Associations between altered FCS and clinical features. All *p*-values are uncorrected.

|  | lFCS | | | | sFCS | | | |
| --- | --- | --- | --- | --- | --- | --- | --- | --- |
| MNI | 30 -12 -33 | | -27 -3 -33 | | 39 48 27 | | -15 -36 54 | |
|  | r | *p* | r | *p* | r | *p* | r | *p* |
| Total Y-BOCS | -0.03 | 0.734 | 0.01 | 0.915 | -0.03 | 0.769 | 0.04 | 0.706 |
| Obsession | -0.08 | 0.438 | -0.01 | 0.943 | -0.04 | 0.723 | 0.07 | 0.470 |
| Compulsion | -0.03 | 0.770 | -0.04 | 0.664 | 0.003 | 0.980 | -0.04 | 0.717 |
| HAMD | 0.11 | 0.270 | 0.05 | 0.604 | 0.03 | 0.804 | 0.005 | 0.962 |
| HAMA | 0.06 | 0.572 | -0.04 | 0.687 | 0.06 | 0.541 | -0.04 | 0.671 |
| Onset age | 0.14 | 0.168 | 0.14 | 0.176 | 0.06 | 0.538 | -0.04 | 0.700 |
| Illness duration | 0.14 | 0.167 | 0.14 | 0.180 | 0.06 | 0.554 | -0.04 | 0.686 |
